# How both positive and burdensome caregiver experiences are associated with care recipient cognitive performance: Evidence from the National Health and Aging Trends Study and National Study of Caregiving

**DOI:** 10.3389/fpubh.2023.1130099

**Published:** 2023-02-13

**Authors:** Alexandra M. Wennberg, Loretta R. Anderson, Annachiara Cagnin, Lenis P. Chen-Edinboro, Lorenzo Pini

**Affiliations:** ^1^Unit of Epidemiology, Institute of Environmental Medicine, Karolinska Institutet, Stockholm, Sweden; ^2^Program in Gerontology, Department of Epidemiology and Public Health, University of Maryland, Baltimore, MD, United States; ^3^Department of Neuroscience (DNS) and Padova Neuroscience Center, University of Padova, Padova, Italy; ^4^School of Health and Applied Human Sciences, University of North Carolina Wilmington, Wilmington, NC, United States; ^5^Padova Neuroscience Center, University of Padova, Padova, Italy

**Keywords:** caregiver, cognition, dementia, positive emotion, care recipient

## Abstract

**Introduction:**

Being an informal caregiver to a person with chronic disease, including persons living with dementia (PLWD), is a big role to take on and many caregivers experience both substantial burden and emotional reward related to caregiving. Care recipient factors (e.g., behavioral symptoms) are associated with caregiver experience. However, the relationship between caregiver and care recipient is bidirectional, so it is likely that caregiver factors impact the care recipient, though few studies have investigated this.

**Methods:**

In the 2017 round of the National Health and Aging Trends Study (NHATS) and National Study of Caregiving (NSOC), we studied 1,210 care dyads-−170 PLWD dyads and 1,040 without dementia dyads. Care recipients completed immediate and delayed word list memory tasks, the Clock Drawing Test, and a self-rated memory rating, while caregivers were interviewed about their caregiving experiences using a 34-item questionnaire. Using principal component analysis, we created a caregiver experience score with three components—Practical Care Burden, Positive Care Experiences, and Emotional Care Burden. We then investigated the cross-sectional association between caregiver experience components and care recipient cognitive test performance using linear regression models adjusted for age, sex, education, race, and depressive and anxiety symptoms.

**Results:**

Among PLWD dyads, a higher caregiver Positive Care Experiences score was associated with better care recipient performance on the delayed word recall (B = 0.20, 95% CI 0.05, 0.36) and Clock Draw (B = 0.12, 95% CI 0.01, 0.24) tests while higher Emotional Care Burden score was associated with worse self-rated memory score (B = −0.19, 95% CI −0.39, −0.003). Among participants without dementia, higher Practical Care Burden score was associated with poorer care recipient performance on the immediate (B = −0.07, 95% CI −0.12, −0.01) and delayed (B = −0.10, 95% CI −0.16, −0.05) word recall tests.

**Discussion:**

These findings support the concept that caregiving is bidirectional within the dyad and that positive variables can positively impact both members of the dyad. This suggests that caregiving interventions should target the caregiver and recipient both individually and as a unit, with the goal of holistically improving outcomes for both.

## Introduction

Informal caregiving is becoming increasingly common, particularly as the population ages. Aging is associated with the accumulation of comorbidities that reduce individuals' ability to perform activities of daily living and necessitate caregiving (1), and many of these individuals rely on informal care. In 2020, 53 million adults in the United States identified as informal caregivers, which is a 22% increase from 2015 (2). Caregiving can be challenging and multi-faceted, including managing practical (e.g., feeding, bathing) as well as emotional and mental (e.g., depression, anxiety, hallucinations) needs of the care recipient. One-third of caregivers care for someone with memory problems, including, but not limited to persons living with dementia (PLWD) (2). Dementia caregivers often must also manage a PLWD's behavioral (e.g., irritability, lack of inhibition) and cognitive (e.g., memory and attentional deficits) changes over years or decades (3). Overall, the demands of caregiving can be challenging and associated with burden and stress; however, caregiving can also be associated with positive experiences, including feeling closer to the care recipient and a sense of meaning (4).

Physical, emotional, financial, and social caregiving burden is associated with negative impacts on physical and mental health, personal and social life, and overall wellbeing (5). Caregivers are at high risk of burnout, depression, cardiovascular disease, hypertension, kidney disease, obesity, polypharmacy, mortality, and developing dementia themselves (6). Simultaneously, they experience social isolation and financial burden (7, 8). Much of caregiving research has focused on the burden and negative impacts of caregiving and led to the conceptualization of the caregiver as “the hidden patient” (9, 10). However, caregiving can also be rewarding and the positive experiences associated with it have been linked with better health outcomes for caregivers (11). For example, we recently showed that among older caregivers, positive emotions associated with caregiving are associated with lower odds of frailty and sleep disruption, while aspects of physical and financial caregiving burden were associated with higher odds of frailty in caregivers (12). The caregiving experience is a spectrum and can be influenced, both positively and negatively, by multiple factors. Exploring these experiences in caregiver-care recipient relationships might help to unravel the complexity of this dyadic relationship, prompting new models for intervention targeting the dyad.

Indeed, a few studies have shown that caregiver interventions can have a direct impact on care recipient outcomes (13, 14), supporting the assumption that caregiving is an interrelated, relational paradigm, where each member of the dyad impacts the other (15). Although there is evidence showing that patient behavioral symptoms impact caregiver burden levels (15), few studies in any disease model, but particularly in dementia caregiving dyads, have examined how caregiver factors may impact care recipient outcomes. To that end, we examined the association between caregiver experience and care recipient cognitive test performance in the National Health and Aging Trends Study (NHATS) and National Study on Caregiving (NSOC). We explored these associations in both dyads where the care recipient had dementia and those where the care recipient did not have dementia. This relationship was analyzed through a data-driven approach aimed at capturing positive and negative caregiving aspects (12). Understanding to what extent caregiver experience is related to care recipient outcomes is critical for understanding how to provide support to both caregiver and recipient.

## Methods

### Data

This was a cross-sectional cohort study that used data from the 2017 rounds of NHATS and NSOC, which were connected at the individual level between caregivers and care recipients. Care recipient data came from NHATS, which is a nationally representative study of Medicare beneficiaries aged 65 and older. Participants were selected from 95 counties or groups of counties within the contiguous United States and were oversampled for non-Hispanic Black Americans and the oldest age groups (16). Data has been collected annually since 2011 *via* annual in-home surveys; additionally, participants (or proxies) provide information about health status, living situation, and care status. Care recipients, if able, completed cognitive testing at each round of the in-person interview conducted by trained data collectors. Caregiver data came from NSOC, which is a periodic study that gathers data on the caregiving experience *via* a phone interview. NSOC participants were identified by the care recipient (or proxy) as someone providing care (e.g., mobility, self-care, household activities, or medical care management) to a NHATS participant, and up to five caregivers were identified for each participant. If the NHATS participant had more than five caregivers, five were selected at random for participation in NSOC. NSOC data was collected in 2011, 2015, and 2017; we chose the 2017 cohort of NSOC, as it is the most recent cohort of caregivers available.

NHATS and NSOC are funded by the National Institute on Aging (U01AG032947) and conducted by the Johns Hopkins Bloomberg School of Public Health. Participation was voluntary and all participants provided written informed consent. NHATS and NSOC were approved by the Johns Hopkins Bloomberg School of Public Health Institutional Review Board.

### Participants

The first round of NHATS (2011) initially included 8245 participants, sampled for national representativeness. In 2014, NHATS resampled for a replenishment sample to compensate for participants who had died or were lost to follow up, leading to a renewed sample of 8334. Details on sampling are available in published white papers (16, 17). In 2017, there were 1947 NHATS participants eligible (i.e., receiving informal care) to be matched to NSOC. This study included 1,210 NHATS participants both with and without dementia who were being cared for by an informal caregiver. Caregivers of the NHATS participants were invited to be part of the NSOC study. In 2017, 4,131 caregivers were eligible to be part of the study and 2,361 were interviewed (18). In the present analyses, the caregiving data came from 1,210 NSOC participants who acted as caregivers. This led to a total sample size of 1,210 dyads with 2,420 matched caregivers and care recipients (Supplementary Figure 1). NSOC took place in 2011, 2015, and 2017.

### Independent variable: Caregiver experience

In NSOC, caregivers completed an extensive telephone interview about their duties and feelings about caregiving with further details provided in previous publications [e.g., (19)]. Caregivers were asked questions relating to practical care (e.g., helping the care recipient with personal care, transportation), the positive aspects of caregiving (e.g., whether the caregiver enjoys being with the care recipient, whether caregiving makes the caregiver feel more confident in their abilities), and emotional burden of caregiving (e.g., whether caregiving felt like too much or exhausting) (Table 1). Caregivers answered questions about whether they performed a duty and how difficult it was for them to perform it on a Likert-scale with 1 being “a little difficult” and 5 being “very difficult.” We included 34 questions and consolidated them using principal component analysis (PCA) to reduce their dimensionality, using previously published methods (12).

**Table 1:** Caregiver experience questions with principal component analysis factor loadings.

	**Practical care burden**	**Positive care experiences**	**Emotional care burden**
How much do you enjoy being with care recipient (CR)?	0.0700	0.3624	0.0393
How much does CR argue with you?	0.1143	0.1633	0.1731
How much does CR appreciate what you do for them?	0.0896	0.2938	0.0133
How often does the CR get on your nerves?	0.1506	0.2323	0.164
Has helping CR has made you more confident about your abilities?	0.0195	0.3957	−0.1662
Has helping CR has taught you to deal with difficult situations?	−0.0313	0.3416	−0.1714
Has helping CR has brought you closer to them?	0.0587	0.4447	−0.0612
Helping CR has given you satisfaction that they are/were well cared for?	0.0298	0.3600	−0.0668
Is/was helping CR financially difficult for you?	0.1583	−0.0900	0.0885
If yes, how financially difficult?	0.1585	−0.085	0.0977
Is/was helping CR emotionally difficult for you?	0.2226	0.0776	−0.0585
If yes, how emotionally difficult?	0.2268	0.1278	0.0182
Is/was helping CR physically difficult for you?	0.1916	−0.0718	−0.0484
If yes, how physically difficult?	0.1921	−0.0665	−0.0095
If you helped CR with chores, how difficult was it for you?	0.2347	−0.066	−0.116
If you helped CR with shopping, how difficult was it for you?	0.2393	−0.0446	−0.126
If you helped CR with ordering prescriptions, how difficult was it for you?	0.1996	−0.1001	−0.158
If you helped CR with bills, how difficult was it for you?	0.2265	−0.0458	−0.1207
If you helped CR with personal care, how difficult was it for you?	0.2312	−0.0598	−0.0742
If you helped CR with getting around their home, how difficult was it for you?	0.2347	−0.0465	−0.0968
If you helped CR by driving them, how difficult was it for you?	0.2205	−0.0067	−0.1207
If you helped CR with transportation, how difficult was it for you?	0.0901	−0.0115	−0.0639
If you helped CR with medications, how difficult was it for you?	0.2247	−0.0655	−0.1823
If you helped CR with managing exercise/diet, how difficult was it for you?	0.1936	−0.1291	−0.0678
If you helped CR with communicating with care providers, how difficult was it for you?	0.2032	0.0007	−0.1165
If you helped CR with coordinating medical care, how difficult was it for you?	0.2245	−0.0251	−0.1133
If you helped CR with managing health insurance, how difficult was it for you?	0.1646	−0.0389	−0.0952
If you helped CR with transitioning post–hospital care, how difficult was it for you?	0.1774	−0.0284	−0.0813
How much has your family disagreed over the details of CR's care?	0.0775	0.0634	0.2774
Do you think you do more than your fair share, less than your fair share, or a fair amount?	0.0990	0.0243	0.3662
Are you exhausted at night?	0.1645	−0.0169	0.3169
Is care more than you can handle?	0.1726	0.0255	0.3614
Do you feel you have no time for yourself?	0.1346	0.0002	0.3804
Do you feel as soon as you get a routine going, CR's needs change?	0.1519	0.0017	0.2889

Non-binary questions ranged from 1 to 5 on a Likert scale and were reversed in some cases to make them consistent with each other.

### Dependent variable: Care recipient cognitive test performance

The cognitive assessment included the immediate and delayed word recall test from the CERAD battery (20). This 10-item word-list memory task asks participants to recall the words immediately after they are presented and again after a delay of approximately 5 min. They also completed the Clock Drawing Test (21), a test of executive function (22), in which participants have 120 s to draw a clock face with the time reading “10 after 11.” The test was scored according to standard criteria ranging from 0 (“not recognizable as a clock) to 5 (“accurate depiction of a clock) (23). Participants additionally rated their memory from 1 (“excellent”) to 5 (“poor”). Care recipients who did not complete cognitive testing were not included in the analyses (Supplementary Figure 1).

### Covariates

Participants or proxies provided information on sex; age group (65–69 years, 70–74 years, 75–79 years, 80–84 years, 85–89 years, and 90+ years); race (White, non-Hispanic; Black, non-Hispanic, Hispanic; other); and education (coded as <high school, high school degree, >high school). Depressive symptoms were assessed with short forms of the Patient Health Questionnaire (PHQ-2) (24) and Generalized Anxiety Disorder Scale (GAD-2) (25).

### Statistical analysis

All analyses were conducted using round-specific survey weights to create nationally representative estimates. This weighting procedure also accounts for the differential probabilities of selection and non-response. NHATS participants or proxy respondents self-reported whether a doctor had ever diagnosed them with dementia (yes/no), so based on this data, we *a priori* stratified participants into PLWD and persons without dementia.

To create the caregiver experience scores, we included the 34 caregiver experience questions in the PCA (Table 1). Three components were identified: Practical Care Burden, Positive Care Experiences, and Emotional Care Burden (described below). This approach was previously used by our group with the purpose of finding different components of caregivers' aspects through a data-driven manner and describing the caregiver experience in a low dimensional space (12). This approach allowed us to define three main components, explaining more than 46% of the variance.

Demographic data from care recipients with dementia were compared to care recipients without dementia using chi-square tests for categorical responses and *t*-tests for continuous responses. We then used survey-weighted multivariable-adjusted linear regression models to investigate the association between caregiver experience component scores (i.e., score on Practical Care Burden, Positive Care Experiences, and Emotional Care Burden components) and their care recipient's cognitive test performance. Model 1 was adjusted for age group and sex. Model 2 was additionally adjusted for education level, race, and depression and anxiety symptoms.

In sensitivity analyses, we restricted the models to only those who lived in community settings (*n* = 1,005), which is relevant because it may be that informal caregivers to individuals living in the community may have to take on more responsibilities, particularly relating to practical caregiving tasks. Conversely, people with more severe dementia may be more likely to live in long-term care institutions. Further, we explored whether the relationship between the caregiver and the care recipient (e.g., spouse, child) affected the observed associations, both as a confounder and effect modifier. All analyses were conducted using Stata 16.0 (StataCorp, College Station, TX).

## Results

In our analyses, among NHATS participants with (*n* = 170) and without (*n* = 1,040) dementia who had an informal caregiver and did not have any missingness on tests of cognitive function or the covariates included in the models, approximately 32.9% were men and most were aged 80 years and older [80–85 (23.7%), 85–89 (20.7%), or 90+ (22.0%)] (Table 2). Most participants were White (67.0%) and had a greater than high school level of education (56.5%). PLWD were older, a greater proportion were men, and they had greater anxiety symptomatology.

**Table 2:** Care recipient characteristics comparing those with and without dementia, *N* (%) or mean (SD) for PHQ-2 and GAD-2.

	**All** ** (*n* = 1,210)**	**PLWD** ** (*n* = 170)**	**Without dementia (*n* = 1,040)**	***P***
Men	398 (32.9)	74 (43.5)	324 (32.9)	0.001
Age group				0.005
65 to 69	54 (4.5)	4 (2.4)	50 (4.8)	
70 to 74	147 (12.2)	8 (4.7)	139 (13.4)	
75 to 79	205 (16.9)	24 (14.1)	181 (17.4)	
80 to 85	287 (23.7)	49 (28.8)	238 (22.9)	
85 to 89	251 (20.7)	43 (25.3)	208 (20.0)	
90+	266 (22.0)	42 (24.7)	224 (21.5)	
Race				0.854
White, non-hispanic	792 (67.0)	115 (67.6)	677 (65.1)	
Black, non-hispanic	312 (26.4)	42 (24.7)	270 (26.0)	
Hispanic	58 (4.9)	2 (1.2)	19 (1.8)	
Other	21 (1.8)	7 (4.1)	51 (4.9)	
Education				0.826
< HS	58 (7.5)	9 (5.3)	49 (4.7)	
HS grad	281 (36.1)	38 (22.4)	243 (23.4)	
>HS	440 (56.5)	56 (32.9)	384 (36.9)	
PHQ-2	1.08 (1.63)	1.29 (1.74)	1.04 (1.61)	0.072
GAD-2	1.18 (1.71)	1.63 (2.05)	1.11 (1.64)	< 0.001
Relationship with caregiver				0.262
Spouse/partner	406 (33.6)	64 (37.6)	342 (32.9)	
Child/grandchild	685 (56.6)	92 (54.1)	593 (47.0)	
Sibling/sibling-in-law	37 (3.1)	7 (4.1)	30 (2.9)	
Other	82 (6.8)	7 (4.1)	75 (7.2)	

HS, high school; PHQ-2, Patient Health Questionnaire; GAD-2, Generalized Anxiety Disorder questionnaire.

From the PCA analysis, based on the Eigenvalues (Supplementary Table 1) and the screeplot (Supplementary Figure 2), we included three components. The 34 question items and the three components are shown in Table 1. Based on the factor loadings and pattern of each component, we named the components “Practical Care Burden,” which was characterized by items about the Practical Care Burden a caregiver provides (e.g., medication management, transportation). The second component we named “Positive Care Experiences,” which was defined by positive experiences with care (e.g., enjoyment being with the care recipient, feelings of closeness to them). The final component we called “Emotional Care Burden,” because it was defined by feelings such as the caregiving being “too much” or being “more than you can handle.” Comparing scores on these components, caregivers who cared for a PLWD had a significantly higher score on the Practical Care Burden component, compared to caregivers who cared for someone who did not have dementia (Supplementary Table 2). There were no differences on the Positive Care Experiences or Emotional Care Burden components.

In linear regression models, we investigated the association between caregiver burden, as defined by these three components, and cognitive performance in the care recipient. Among all participants, in models adjusted for age and sex, higher score on the Practical Care Burden component was associated with the care recipient having poorer performance on the immediate word recall (B = −0.09, 95% CI −0.13, −0.06), delayed word recall (B = −0.12, 95% CI −0.16, −0.09), and Clock Draw (B = −0.04, 95% CI −0.07, −0.008) tests (Table 3). Higher Practical Care Burden score was associated with worse self-rated memory among care recipients (B = 0.03, 95% CI 0.01, 0.06). After additionally adjusting for education, race, and depression and anxiety symptoms, the associations remained for immediate (B = −0.10, 95% CI −0.15, −0.05) and delayed (B = −0.13, 95% CI −0.18, −0.08) word recall performance. In Model 1, a higher score on Positive Care Experiences was associated with higher immediate word recall score (B = 0.06, 95% CI 0.0002, 0.12).

**Table 3:** Association between caregiver experience and care recipient cognitive test performance.

	**Practical care burden**		**Positive care experiences**		**Emotional care burden**	
	**B (95% CI)**	***p***	**B (95% CI)**	***p***	**B (95% CI)**	***p***
**Model 1**						
Rate memory	0.03 (0.01, 0.06)	0.004	−0.04 (−0.09, 0.002)	0.061	−0.03 (−0.10, 0.04)	0.438
Immediate word recall	−0.09 (−0.13, −0.06)	< 0.001	0.06 (0.0002, 0.12)	0.049	−0.02 (−0.11, 0.07)	0.669
Delayed word recall	−0.12 (−0.16, −0.09)	< 0.001	0.005 (−0.07, 0.08)	0.886	0.009 (−0.09, 0.10)	0.844
Clock Draw	−0.04 (−0.07, −0.008)	0.017	0.03 (−0.01, 0.08)	0.154	−0.05 (−0.12, 0.020)	0.181
**Model 2**						
Rate memory	0.005 (−0.03, 0.04)	0.760	−0.008 (−0.06, 0.04)	0.743	−0.03 (−0.10, 0.05)	0.434
Immediate word recall	−0.10 (−0.15, −0.05)	< 0.001	−0.02 (−0.10, 0.07)	0.697	0.005 (−0.11, 0.11)	0.930
Delayed word recall	−0.13 (−0.18, −0.08)	< 0.001	−0.006 (−0.11, 0.10)	0.910	0.03 (−0.09, 0.16)	0.585
Clock Draw	−0.04 (−0.08, 0.005)	0.079	0.007 (−0.05, 0.07)	0.812	−0.03 (−0.13, 0.06)	0.456

Model 1 adjusted for age and sex. Model 2 adjusted for age, sex, education, race, depression, and anxiety symptoms.

Model 1: Population size = 114,831,880; Rate memory, *n* = 1,178; Immediate word recall, *n* = 1,210; Delayed word recall, *n* = 1,207; Clock Draw, *n* = 1,192. Model 2: Rate memory, *n* = 730, pop *n* = 111,814,456; Immediate word recall, *n* = 751, pop *n* = 111,735,104; Delayed word recall, *n* = 748, pop *n* = 111,735,104; Clock Draw, *n* = 742, pop *n* = 111,780,037.

Among PLWD, in the fully adjusted model, higher score on Positive Care Experiences was associated with better care recipient performance on the delayed word recall (B = 0.20, 95% CI 0.05, 0.36) and Clock Draw (B = 0.12, 95% CI 0.01, 0.24) tests (Table 4). Contrastingly, higher score on the Emotional Care Burden component was associated with worse self-rated memory (B = −0.19, 95% CI −0.39, −0.003).

**Table 4:** Associaiton between caregiver experience and care recipient cognitive test performance among care recipients living with dementia.

	**Practical care burden**		**Positive care experiences**		**Emotional care burden**	
	**B (95% CI)**	***p***	**B (95% CI)**	***p***	**B (95% CI)**	***p***
**Model 1**						
Rate memory	−0.02 (−0.08, 0.04)	0.503	0.07 (−0.05, 0.18)	0.271	−0.02 (−0.15, 0.11)	0.769
Immediate word recall	−0.07 (−0.15, 0.02)	0.126	0.06 (−0.09, 0.21)	0.443	0.09 (−0.21, 0.40)	0.543
Delayed word recall	−0.04 (−0.11, 0.03)	0.283	0.08 (−0.05, 0.21)	0.234	0.13 (−0.16, 0.42)	0.367
Clock Draw	−0.05 (−0.13, 0.02)	0.165	0.11 (−0.002, 0.23)	0.054	−0.13 (−0.34, 0.08)	0.217
**Model 2**						
Rate memory	−0.05 (−0.13, 0.03)	0.235	0.11 (−0.02, 0.24)	0.096	−0.19 (−0.39, −0.003)	0.047
Immediate word recall	−0.10 (−0.22, 0.02)	0.098	0.09 (−0.06, 0.25)	0.238	0.004 (−0.34, 0.35)	0.980
Delayed word recall	−0.09 (−0.20, 0.02)	0.098	0.20 (0.05, 0.36)	0.012	−0.006 (−0.38, 0.37)	0.976
Clock Draw	−0.05 (−0.16, 0.05)	0.309	0.12 (0.01, 0.24)	0.032	−0.17 (−0.43, 0.10)	0.219

Model 1 adjusted for age and sex. Model 2 adjusted for age, sex, education, race, depression, and anxiety symptoms. Model 1: Rate memory, *n* = 151, pop *n* = 106,892,009; Immediate word recall, *n* = 170, pop *n* = 108,383,485; Delayed word recall, *n* = 169, pop *n* = 108,383,485; Clock Draw, *n* = 160, pop *n* = 108,383,485. Model 2: Rate memory, *n* = 86, pop *n* = 94,911,991; Immediate word recall, *n* = 99, pop *n* = 105,123,282; Delayed word recall, *n* = 98, pop *n* = 105,123,282; Clock Draw, *n* = 95, pop *n* = 105,148,641.

Among participants without dementia, in both Models 1 and 2, higher score on the Practical Care Burden component was associated with poorer care recipient performance on the immediate (Model 1: B = −0.07, 95% CI −0.10, −0.03; Model 2: B = −0.07, 95% CI −0.12, −0.01) and delayed (Model 1: B = −0.10, 95% CI −0.14, −0.06; Model 2: B = −0.10, −0.16, −0.05) word recall tests (Table 5).

**Table 5:** Association between caregiver experience and care recipient cognitive test performance among care recipients without dementia diagnosis.

	**Practical care burden**		**Positive care experiences**		**Emotional care burden**	
	**B (95% CI)**	***p***	**B (95% CI)**	***p***	**B (95% CI)**	***p***
**Model 1**						
Rate memory	0.02 (−0.004, 0.05)	0.091	−0.04 (−0.10, 0.01)	0.118	−0.02 (−0.09, 0.05)	0.550
Immediate word recall	−0.07 (−0.10, −0.03)	0.001	0.04 (−0.01, 0.09)	0.151	−0.05 (−0.14, 0.05)	0.337
Delay word recall	−0.10 (−0.14, −0.06)	< 0.001	−0.03 (−0.11, 0.05)	0.478	−0.02 (−0.12, 0.08)	0.707
Clock Draw	−0.03 (−0.06, 0.01)	0.153	0.01 (−0.03, 0.06)	0.552	−0.04 (−0.11, 0.03)	0.296
**Model 2**						
Rate memory	−0.005 (−0.04, 0.03)	0.778	−0.02 (−0.08, 0.04)	0.486	−0.02 (−0.10, 0.06)	0.623
Immediate word recall	−0.07 (−0.12, −0.01)	0.014	−0.05 (−0.13, 0.03)	0.238	−0.002 (−0.10, 0.10)	0.971
Delayed word recall	−0.10 (−0.16, −0.05)	0.001	−0.05 (−0.15, 0.06)	0.356	0.02 (−0.10, 0.14)	0.721
Clock Draw	−0.02 (−0.06, 0.03)	0.471	−0.02 (−0.08, 0.04)	0.582	−0.02 (−0.12, 0.07)	0.613

Model 1 adjusted for age and sex. Model 2 adjusted for age, sex, education, race, depression, and anxiety symptoms.

Model 1: Rate memory, *n* = 1,027, pop *n* = 114,831,880; Immediate word recall, *n* = 1,040, pop *n* = 114,831,880; Delayed word recall, *n* = 1,038, pop *n* = 114,831,880; Clock Draw, *n* = 1,032, pop *n* = 114,831,880. Model 2: Rate memory, *n* = 644, pop *n* = 112,148,522; Immediate word recall, *n* = 652, pop *n* = 112,100,582; Delayed word recall, *n* = 650, pop *n* = 112,100,582; Clock Draw, *n* = 647, pop *n* = 112,100,582.

In the sensitivity analyses restricted to those who lived in community settings (*n* = 1,005), we did not find any evidence of differences in the associations. This is notable because it indicates that the relationship between caregiver and care recipient is important even if the care recipient is not living in the home. In further analyses, exploring whether relationship between the caregiver and recipient impacted the association, we found it did not. We conducted additional sensitivity analyses adjusting for presence of caregiver health conditions (heart attack, heart disease, hypertension, arthritis, osteoporosis, diabetes, lung disease, and serious hearing and/or vision impairment), as these may be associated to caregiver experience. We found adjusting for presence of caregiver health conditions did not impact the strength or direction of the observed associations (results not shown).

## Discussion

In this study of 1,201 dyads of older adults and their informal caregivers, the core result is the relationship between different facets of caregiver experience with care recipient cognitive performance depending on whether the care recipient has dementia or not. In dyads where the care recipient had dementia, positive feelings associated with caregiving were positively associated with care recipients performing better on tests of memory and executive function. In dyads where the care recipient did not have dementia, higher scores on the Practical Care Burden component were associated with poorer immediate and delayed word recall performance.

Past research in caregiving, particularly dementia caregiving, has shown that care recipient symptoms (e.g., behavioral symptoms) impact the caregiver (15). Specifically, care recipient depressive symptoms, accusatory or aggressive behavior, non-threatening psychotic behavior, and difficult behavior are all associated with caregiver depression. However, a dyadic caregiving relationship is bidirectional, so it is reasonable that caregiver wellbeing would also impact the care recipient. Most of the research into how caregiver experience and characteristics impact the care recipient has been done in dyads where the care recipient has heart failure. These studies show that greater caregiver burden is associated with worse patient symptoms and quality of life and higher risk of clinical events (26). Studies in neurodegenerative disease, including amyotrophic lateral sclerosis (ALS) and dementia, have shown similar findings. In 33 ALS care dyads, caregiver burden was associated with greater apathy, disinhibition, and executive dysfunction in the care recipient (27). To our knowledge, only one study has previously examined the association between caregiver experience and care recipient outcomes in dementia care dyads. In a sample of 1,201 dyads, it was found that greater caregiver burden measured with questionnaires was associated with poorer care recipient cognitive performance, as measured by the Mini Mental State Examination, which is a global screening tool and less specific measure of cognition than the tests used here (28). Similar to the present findings, the association did not differ by dyad relationship (i.e., spouse, child, etc.,). These findings lend support to the results from this study, suggesting that there is a bidirectional effect between caregiver and care recipient. This evidence then suggests that caregiver interventions would positively impact not just caregiver wellbeing, but care recipient wellbeing, too.

Both caregiver and recipient appraisal of the situation and coping strategies affect both caregiver and recipient adjustment (29). A study that investigated the association between caregiving demands and burden found that this relationship was significantly mediated by caregivers' perceived injustice (e.g., feeling that their life will never be the same or that the situation is unfair) (30). This suggests that greater caregiving demands (e.g., Practical Care Burden) are associated with greater perceived injustice, and subsequently, poorer wellbeing. Care recipient behaviors can impact resentment; however, forgiveness can be fostered through reframing and reappraisal (30, 31). Bidirectional appraisal and coping are iterative processes that change over time based on multiple factors. Dyadic coping strategies that acknowledge the disease, stressors, and change in relationship can reduce burden for both members of the dyad (32). Moving beyond this, caregiving research, particularly dementia caregiving, has centered on a “burden of care” model, which focuses on failure. Recently, however, there has been a paradigm shift toward models of care that bolster sustainability by harnessing caregiver potential for resilience. This approach may be the most effective for improving and preventing health outcomes in all members of the care network (33). While caregiving interventions that target the caregiver are useful, because of this cyclical and bidirectional relationship, interventions that include both caregiver and recipient—individually and as a unit—will likely be the most impactful.

Interventions in cancer and heart failure dyads showing improvements for both caregiver and care recipient also suggests that this association can be positively altered (34, 35). However, few studies (13, 14) have investigated the direct impact of a caregiver intervention on care recipient outcomes in PLWD. These interventions were tailored and incorporated both elements of support and education. Interventions that included caregivers and recipients with dementia together show that care recipients improve on measures of depression and neuropsychiatric symptoms over the course of a year. Additionally, caregivers improved on measures of functional ability, depression, neuropsychiatric symptoms, and burden (14). Additional studies have also shown improvement in care recipient depression, as well as functioning and quality of life (13). It would seem then that dyadic, holistic intervention strategies may have the dual-level benefit of improving outcomes in both caregiver and recipient, and then, subsequently, these improvements will positively feedback into further improvements.

Further, communication and interaction training can provide dyads with tools to repair harm in relationships and address changes in family dynamics (36). Providing training with engaged listening and use of positive language can work to improve the strain in the changing relationship. Moreover, using role-playing to train the dyad in how to meaningfully engage in activities together, by structuring them into steps so that both members of the dyad may meaningfully participate can be helpful. Bonding activities which once again allow the dyad to enjoy time together and connect, including activities that focus on reminiscence, orientation, and rerolling, can be beneficial to both members of the care dyad (37, 38).

This study has several strengths, including a large, nationally representative sample. However, the findings from this study showed be considered in view of its limitations. First, the study is cross-sectional, so causality cannot be determined. However, here we examined the association between caregiver experience and care recipient cognitive performance. Although there is substantial literature showing that care recipient behavioral and neuropsychiatric symptoms impact caregiver burden, a recent systematic review showed that there is no suggestion that care recipient cognitive level impacts caregiver burden (39). Therefore, the observed associations here are likely in the direction of caregiver to recipient, in this, overall, bidirectional relationship; however, longitudinal studies are needed to further investigate the directionality. Second, we were unable to include data on whether some caregivers may have received government insurance-based financial assistance to compensate them for their caregiving role, which may aid in reducing burden caused by financial constraints and thus impact the strength of the observed association. We performed sensitivity analysis restricting to care recipients living in community settings and thus less likely to receive formal assistance compared to care recipients living in institutionalized settings, where caregivers may have fewer day-to-day caregiving responsibilities. However, care recipients in the community may still receive formal care support and caregivers to care recipients in institutionalized settings may still face many caregiving-related challenges. Therefore, we acknowledge that the sensitivity analysis does not entirely address the differences of these circumstances and future work specifically investigating these differences should be conducted. Additionally, dementia was self-reported in this study and although other have used NHATS data to identify participants with possible and probable dementia (22), we wanted to include a more specific PLWD group. However, it may be that by using “gold-standard” dementia diagnosis (e.g., NINCDS-ADRDA) identified by a neurologist, the results could change. Different measures of cognitive ability may also impact the associations observed, though we believe similar trends would be seen. Delayed verbal memory was assessed here, which is the domain most strongly impacted in Alzheimer's disease (40), the most common cause of dementia (41), caregiver experience may be differentially associated with other types of memory (e.g., visuospatial memory) in the care recipient. Finally, particularly regarding the PLWD group, it is unknown what type or stage of dementia they have. Although Alzheimer's disease is the most common cause of dementia, vascular dementia and frontotemporal dementia affect many patients and can present with different behavioral and neuropsychiatric symptoms. Future research should be done to investigate how the associations shown here may change based on dementia type.

## Conclusion

This work stresses the importance of providing holistic care for caregiving dyads and emphasizing positive aspects of caregiving. Decades of evidence have shown that the stress and burden of caregiving is associated with poorer physical and mental health outcomes in caregivers (6). However, it is important to consider that this stress and burden may affect the care recipient, as well. Future work should use longitudinal data to further investigate the directionality and long-term associations between caregiver experience and care recipient outcomes. Most notably, this work shows that the positive elements of caregiving are positively associated with outcomes in PLWD, therefore, harnessing those feelings and perceptions of the caregiving role can beneficially impact both caregiver and recipient. There is the potential to impact outcomes more robustly in both members of the dyad through intervention studies by first understanding the nature of this bidirectional relationship.

## Data availability statement

The original contributions presented in the study are included in the article/Supplementary material, further inquiries can be directed to the corresponding author.

## Ethics statement

The studies involving human participants were reviewed and approved by Johns Hopkins University Institutional Review Board. The patients/participants provided their written informed consent to participate in this study.

## Author contributions

AMW conceived of the study, completed the analysis, and drafted the manuscript. LRA helped to conceive of the study, provided expertise with the data, and helped to write and edit the manuscript. AC provided clinical expertise with regard to caregiver and recipient relationships and cognitive tests and helped to write and edit the manuscript. LPC-E provided guidance with regards to methods and theory and helped to write and edit the manuscript. LP provided analytical support, guidance with regards to methods and theory, and helped to write and edit the manuscript. All authors contributed to manuscript revision, read, and approved the submitted version.
