# Supplementary material for: How both positive and burdensome caregiver experiences are associated with care recipient cognitive performance: Evidence from the National Health and Aging Trends Study and National Study of Caregiving
Source: Front Public Health. 2023 Feb 13;11:1130099. doi: 10.3389/fpubh.2023.1130099 (PMC9969137; doi:10.3389/fpubh.2023.1130099)
Supplement: Supplementary file 1 [file Table_1.docx]

| Supplementary Table 1. Factor loadings from principal component analysis | | | | |
| --- | --- | --- | --- | --- |
| Component | Eigenvalue | Difference | Proportion | Cumulative |
| Comp1 | 11.2539 | 8.42496 | 0.331 | 0.331 |
| Comp2 | 2.82895 | 1.16499 | 0.0832 | 0.4142 |
| Comp3 | 1.66396 | 0.189661 | 0.0489 | 0.4631 |
| Comp4 | 1.4743 | 0.119705 | 0.0434 | 0.5065 |
| Comp5 | 1.35459 | 0.098137 | 0.0398 | 0.5463 |
| Comp6 | 1.25646 | 0.018805 | 0.037 | 0.5833 |
| Comp7 | 1.23765 | 0.225867 | 0.0364 | 0.6197 |
| Comp8 | 1.01179 | 0.096738 | 0.0298 | 0.6495 |
| Comp9 | 0.915047 | 0.047008 | 0.0269 | 0.6764 |
| Comp10 | 0.86804 | 0.074029 | 0.0255 | 0.7019 |
| Comp11 | 0.79401 | 0.052971 | 0.0234 | 0.7253 |
| Comp12 | 0.74104 | 0.005939 | 0.0218 | 0.7471 |
| Comp13 | 0.7351 | 0.110403 | 0.0216 | 0.7687 |
| Comp14 | 0.624697 | 0.035453 | 0.0184 | 0.787 |
| Comp15 | 0.589244 | 0.029335 | 0.0173 | 0.8044 |
| Comp16 | 0.559909 | 0.025746 | 0.0165 | 0.8208 |
| Comp17 | 0.534163 | 0.036764 | 0.0157 | 0.8366 |
| Comp18 | 0.4974 | 0.015409 | 0.0146 | 0.8512 |
| Comp19 | 0.481991 | 0.004745 | 0.0142 | 0.8654 |
| Comp20 | 0.477246 | 0.016018 | 0.014 | 0.8794 |
| Comp21 | 0.461229 | 0.037218 | 0.0136 | 0.893 |
| Comp22 | 0.424011 | 0.005201 | 0.0125 | 0.9054 |
| Comp23 | 0.418811 | 0.032231 | 0.0123 | 0.9178 |
| Comp24 | 0.386579 | 0.017795 | 0.0114 | 0.9291 |
| Comp25 | 0.368785 | 0.011575 | 0.0108 | 0.94 |
| Comp26 | 0.357209 | 0.01468 | 0.0105 | 0.9505 |
| Comp27 | 0.34253 | 0.03614 | 0.0101 | 0.9605 |
| Comp28 | 0.30639 | 0.033612 | 0.009 | 0.9696 |
| Comp29 | 0.272777 | 0.021648 | 0.008 | 0.9776 |
| Comp30 | 0.251129 | 0.037528 | 0.0074 | 0.985 |
| Comp31 | 0.213601 | 0.079136 | 0.0063 | 0.9913 |
| Comp32 | 0.134466 | 0.052395 | 0.004 | 0.9952 |
| Comp33 | 0.08207 | 0.001151 | 0.0024 | 0.9976 |
| Comp34 | 0.080919 | . | 0.0024 | 1 |
