# Supplementary material for: How both positive and burdensome caregiver experiences are associated with care recipient cognitive performance: Evidence from the National Health and Aging Trends Study and National Study of Caregiving
Source: Front Public Health. 2023 Feb 13;11:1130099. doi: 10.3389/fpubh.2023.1130099 (PMC9969137; doi:10.3389/fpubh.2023.1130099)
Supplement: Supplementary file 2 [file Table_2.docx]

| Supplementary Table 2. Comparison of caregiver experience component scores by those caring for a person living with dementia vs those without, mean (SD) | | | | | |
| --- | --- | --- | --- | --- | --- |
| Component | Total | PLWD | Without | p |  |
| Practical Care Burden | -0.18 (3.29) | 1.35 (3.53) | -0.43 (3.19) | <0.001 |  |
| Positive Care Experiences | -0.01 (1.67) | -0.07 (1.79) | -0.004 (1.64) | 0.652 |  |
| Emotional Care Burden | -0.04 (1.27) | -0.11 (1.26) | -0.02 (1.28) | 0.397 |  |
