# Supplementary figures and images for: How both positive and burdensome caregiver experiences are associated with care recipient cognitive performance: Evidence from the National Health and Aging Trends Study and National Study of Caregiving
Source: Front Public Health. 2023 Feb 13;11:1130099. doi: 10.3389/fpubh.2023.1130099 (PMC9969137; doi:10.3389/fpubh.2023.1130099)

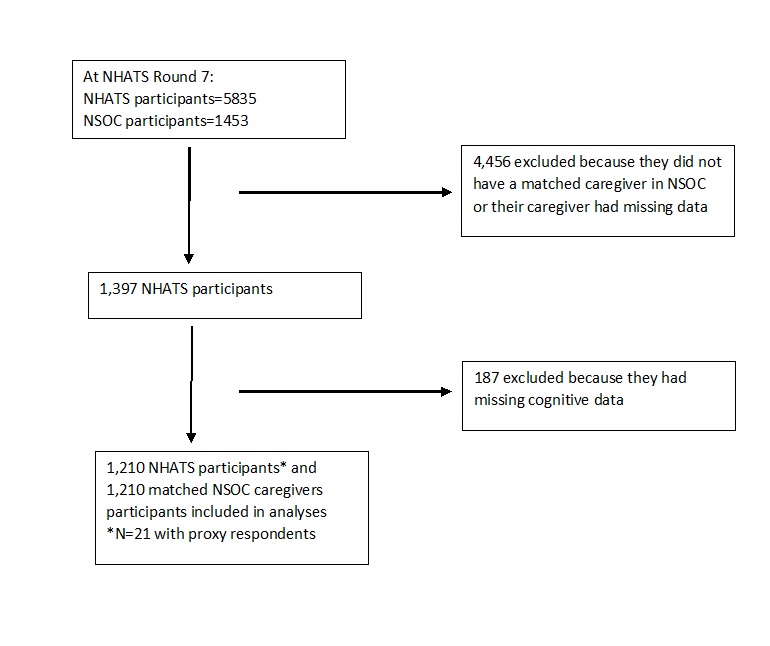

Supplement: Supplementary file 3 [file Image_1.jpg]

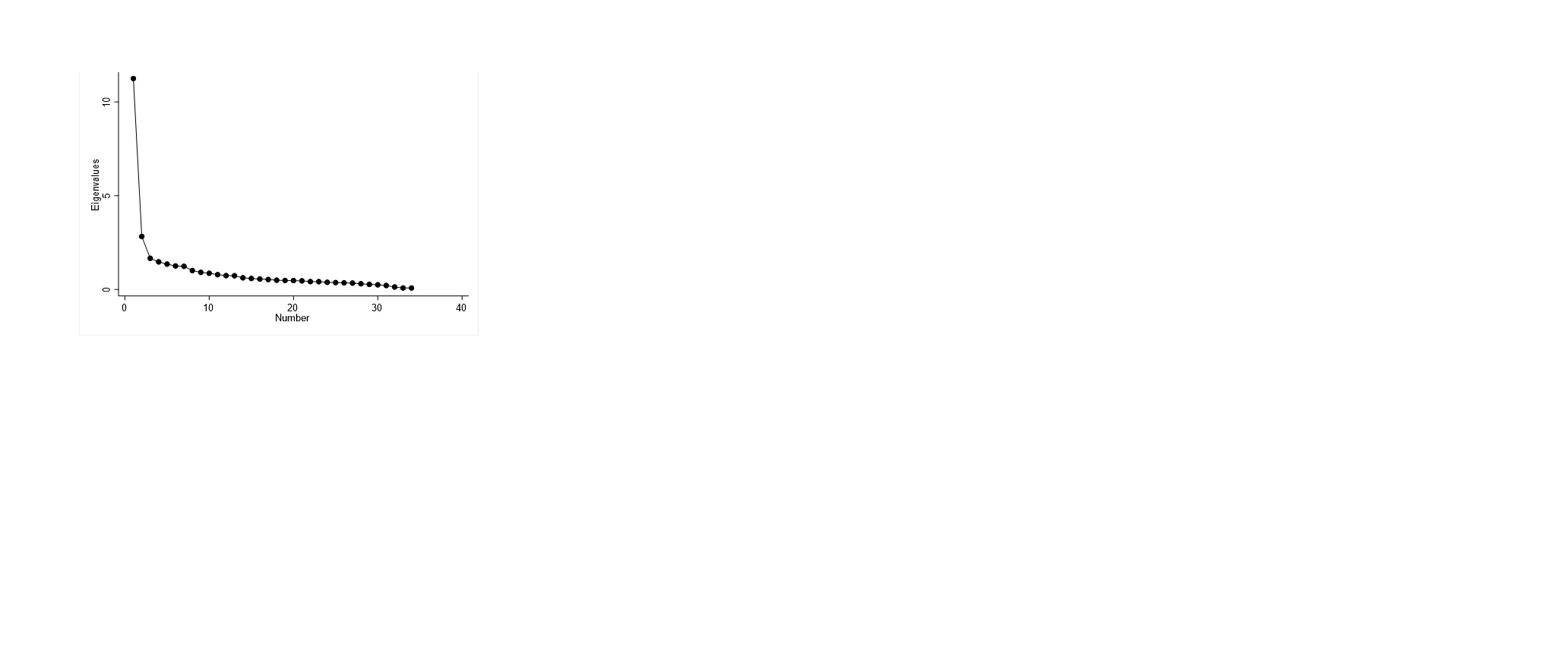

Supplement: Supplementary file 4 [file Image_2.jpg]
